# Reconstructing temporal and spatial dynamics from single-cell pseudotime using prior knowledge of real scale cell densities

**DOI:** 10.1038/s41598-020-60400-z

**Published:** 2020-02-27

**Authors:** Karsten Kuritz, Daniela Stöhr, Daniela Simone Maichl, Nadine Pollak, Markus Rehm, Frank Allgöwer

**Affiliations:** 1 Institute for Systems Theory and Automatic Control, University of Stuttgart, Stuttgart, Germany; 2 Institute of Cell Biology and Immunology, University of Stuttgart, Stuttgart, Germany; 3 Stuttgart Research Center Systems Biology, University of Stuttgart, Stuttgart, Germany

**Keywords:** Cancer, Cell biology, Computational biology and bioinformatics, Systems biology

## Abstract

Modern cytometry methods allow collecting complex, multi-dimensional data sets from heterogeneous cell populations at single-cell resolution. While methods exist to describe the progression and order of cellular processes from snapshots of such populations, these descriptions are limited to arbitrary pseudotime scales. Here we describe MAPiT, an universal transformation method that recovers real-time dynamics of cellular processes from pseudotime scales by utilising knowledge of the distributions on the real scales. As use cases, we applied MAPiT to two prominent problems in the flow-cytometric analysis of heterogeneous cell populations: (1) recovering the kinetics of cell cycle progression in unsynchronised and thus unperturbed cell populations, and (2) recovering the spatial arrangement of cells within multi-cellular spheroids prior to spheroid dissociation for cytometric analysis. Since MAPiT provides a theoretic basis for the relation of pseudotime values to real temporal and spatial scales, it can be used broadly in the analysis of cellular processes with snapshot data from heterogeneous cell populations.

## Introduction

Quantitative single-cell measurements with ten to several thousands of cellular components in large populations provide new opportunities and challenges to study biological processes^1–3^. Since cells within heterogeneous populations span all stages and transitions of a biological process of interest and hence also the dynamics of cellular components, the temporal evolution of cellular signalling events can be inferred from static snapshot data. Several algorithms, such as Wanderlust, Monocle or diffusion pseudotime (DPT), designed to reconstruct cell trajectories, order single-cell data in *pseudotime* – a quantitative measure of progress through a biological process^4–9^. However, these pseudotime trajectories may deviate substantially from the real-time trajectory^10,11^. Alternative approaches attempting to transfer pseudotime to real-time analysis are technically restricted, e.g. limited to cell cycle analysis^12,13^, require specific source data, such as single-cell RNA-seq data^14^, or require computationally expensive estimations of non-identifiable functions^9,15^. A more detailed discussion on attempts to transform pseudotime scales is provided in the Supplementary Information.

**Figure 1:**
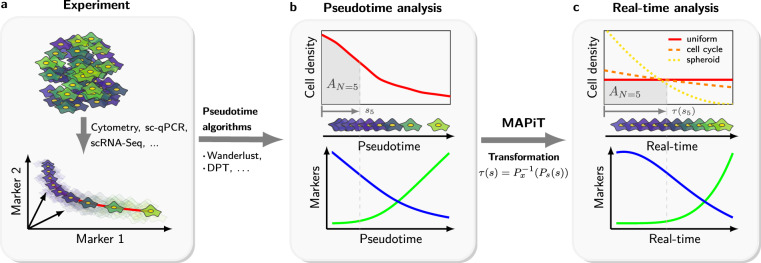
MAPiT deduces process dynamics from single-cell snapshot data. (**a**) Cells from single-cell experiments of a heterogeneous population are ordered on a process manifold in dataspace by pseudotime algorithms. (**b**) Cell density and marker trajectories on pseudotime scale vary with the distance measure used by the pseudotime algorithm and real temporal trajectories cannot be deduced. Cell density, order and trajectories for two markers on pseudotime scale are shown for an exemplary process. As an example pseudotime position of the fifth displayed cell ${s}_{5}$ and associated area under the cell density curve ${A}_{N=5}$ are indicated in gray. (**c**) Nonlinear transformation of pseudotime scale recovers true scale dynamics. MAPiT uses prior knowledge of cell densities on the real scale to transform pseudotime to real time by enforcing equality for the area under the density curves at corresponding points on both scales (gray areas). Cell order and marker trajectories are shown for an exemplary uniform distribution on the real scale. Positions of cells across the cell cycle (dashed, orange) or decreasing number of cells towards the centre of spheroid cultures (dotted, yellow) are other real scale densities.

Here, we developed a measure-preserving transformation of pseudotime into real-time, a MAP of pseudotime into Time, in short MAPiT^16^. MAPiT generalises approaches based on ergodic principles to provide a simple and at the same time universal method to obtain true scale dynamics from pseudotime ordering (Fig. 1). The method makes use of pseudotemporal ordering obtained from trajectory inference algorithms and hence is reliant on the correctness of the provided cell order.

## Results

Common pseudotime algorithms order cells on a pseudotime scale based on a distance metric in the data space, and this metric differs between algorithms^17^. Pseudotime values furthermore strongly depend on the measured cellular components. MAPiT resolves the arbitrariness of pseudotime by nonlinearly transforming pseudotime to the true scale of the process. This is based on a “measure-preserving transformation” which ensures that the area under the curve is conserved when transforming a probability distribution (Materials and Methods). The transformation requires knowledge of the distributions (or cumulative distributions) of cells on both scales (pseudotime and desired scale). Pseudotime values from experimental data can be used to calculate the distribution on the pseudotime scale. In contrast, a priori knowledge of the process of interest must be used to derive the distribution of cells on the desired real-time scale, as we demonstrate in the following examples.

**Figure 2:**
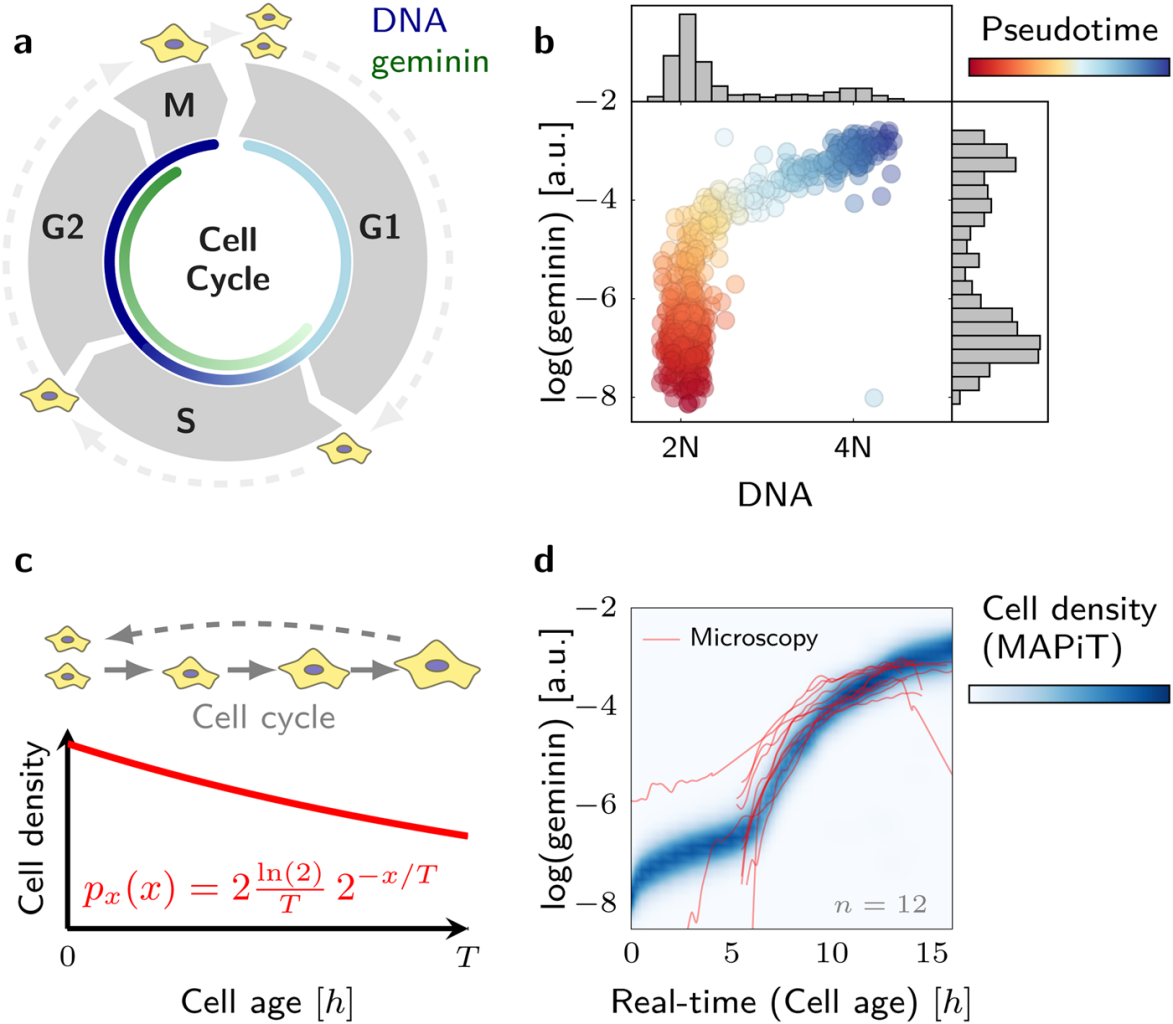
MAPiT recovers cell cycle dynamics. (**a**) Schematic of the cell cycle with geminin expression, a marker for cell cycle progression, starting at the onset of S phase. (**b**) DNA and geminin signals from an unsynchronised population of NCI-H460/geminin cells were used to obtain a pseudotemporal ordering of the population. (**c**) MAPiT employs steady state age distribution of unsynchronised cell populations with cell cycle length $T$. (**d**) Reconstructed temporal profile of the marginal geminin signal density $p(y| x)$ obtained with MAPiT and single-cell trajectories from time-lapse imaging correlate strongly. The geminin intensity, spanning several orders of magnitude, exceeds the detection range in the imaging experiment such that G1 phase cells had signals below the detection limit.

We first applied MAPiT to analyse cell cycle progression. To this end, we used a static flow cytometric measurement of DNA and *mAG-hGeminin (1–110)*, a fluorescent ubiquitination-based cell cycle indicator (Fig. 2a)^13,18^, in unperturbed NCI-H460/geminin cells to reconstruct the kinetics of geminin. The pseudotime obtained with the markers (Fig. 2b) was mapped to the temporal scale on which the the cell cycle progresses, namely the age of a cell (time since cytokinesis). To achieve this, MAPiT derives real-time trajectories from the steady state age distribution^12,13,19^ (Fig. 2c). Temporal trajectories of geminin obtained with MAPiT corresponded excellently to geminin kinetics obtained by single cell time-lapse microscopy (Fig. 2d), as exemplified by the boost in geminin intensity at approximately $7h$, indicating the onset of S-phase. This result therefore highlights the temporal accuracy of the real-time scale obtained by MAPiT for cell cycle analysis based on snapshot flow cytometric data.

**Figure 3:**
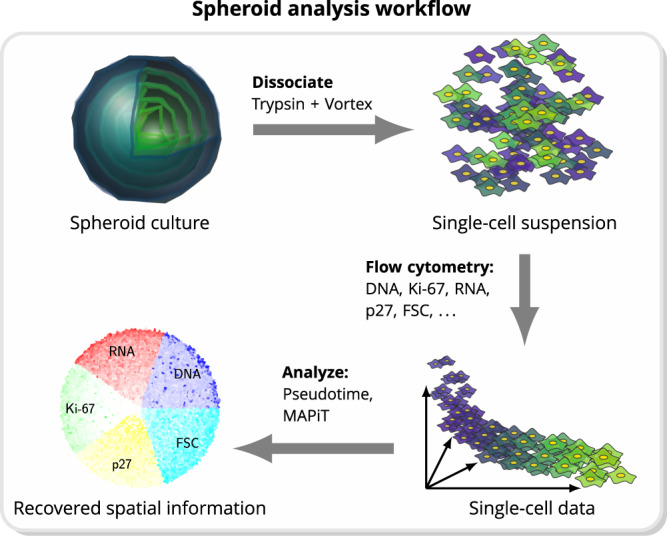
MAPiT recovers spatial positions of cells within spheroids from flow cytometric data. Illustration of spheroid analysis workflow. Individual cells derived from dissociated spheroids were analysed for different markers by flow cytometry. Spatial information can be recovered by applying MAPiT to pseudotime trajectories of measured markers.

**Figure 4:**
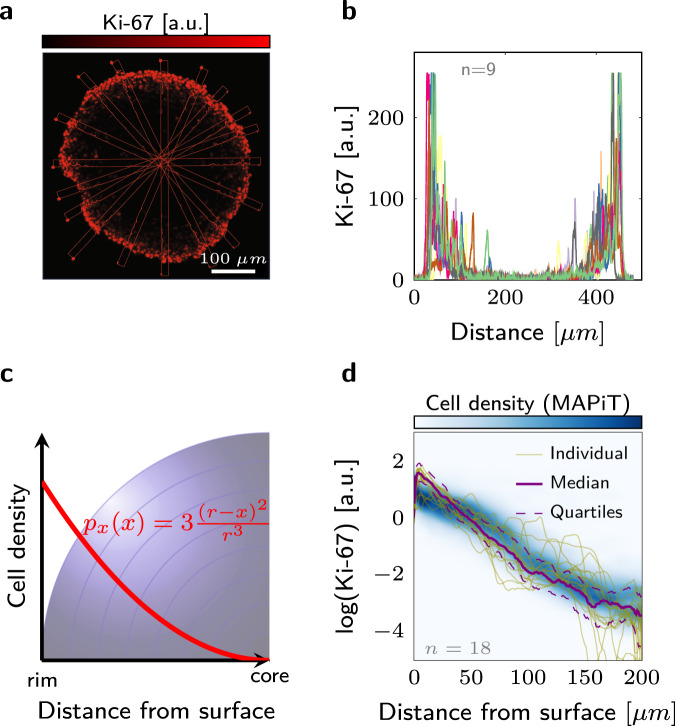
MAPiT derived Ki-67 distribution in an 11-day-old HCT116 cell spheroid. (**a**) Representative spheroid cross-section stained for Ki-67. Rectangles display cross sectional signal intensities for comparison with MAPiT. (**b**) Transversal quantified Ki-67 intensities in spheroid sections (rectangles in (**a**), $n=9$). (**c**) MAPiT employs cell density in radial-symmetric spheroids with radius $r$, of which the derivation is contained in detail in the Materials and Methods. (**d**) Marginal Ki-67 signal density obtained by MAPiT from flow cytometric data (blue) closely match Ki-67 intensity profiles determined microscopically in spheroid cross-sections.

Multicellular spheroids grown from cancer cells are widely used as avascular tumour models^20–22^. As a consequence of nutrient and oxygen deprivation within the spheroids, proliferative cells begin to enclose inner layers of quiescent and necrotic cells, resembling a zonation found in solid tumours^23,24^. Current routine methods to study spatial distributions and patterns of cellular markers are restricted to intact spheroids, technically cumbersome and of limited throughput, since they rely on sequential spheroid fixation, sectioning and imaging procedures. By dissociating tumour spheroids for single cell experiments, spatial information across which cell-to-cell heterogeneities in tumour cells spheroids manifest is lost. We applied MAPiT to study if we can recover spatial scales from flow cytometric measurements of dissociated spheroids in a reliable and robust manner. For our studies, we grew spheroids of HCT116 cells to diameters of approximately $500\ \mu m$. Besides standard light scatter readouts like forward scatter (FSC), indicating cell volume, cells were in addition stained for DNA as measure for cell cycle stage, RNA as indicator for transcriptional activity, Ki-67 as marker for proliferation and p27 as marker for quiescence (Fig. 3). To apply MAPiT to these data, the distribution of cells on the spatial scale had to be taken into account, which in the case of radial symmetry of spheroids is the density of cells in relation to the distance from the spheroid surface (Figs. 4c, S2). In brief, the volume of a spherical shell on the spheroid surface, normalised to the volume of the whole sphere equals the cumulative distribution of cells in relation to the distance from the surface. A more detailed discussion on spheroid volume and related growth rate is provided in the Supplementary Information.

We first validated MAPiT results against images of stained spheroid sections. Following the workflow depicted in Fig. 3, we prepared Ki-67 distributions from 11-day old HCT116 spheroids with MAPiT. Spheroids from the same culture were sliced and stained for Ki-67 as described in Materials and Methods (Fig. 4a). We then quantified transversal signal intensities in the spheroid sections (Fig. 4b). MAPiT indeed recovered the spatial positions of single cells, with the reconstructed Ki-67 distribution correlating excellently with intensity profiles obtained from confocal microscopy of spheroid sections stained for Ki-67 (Fig. 4d).

Next, we examined if MAPiT can also capture the heterogeneity within the different zones in the spheroid, typically a proliferative layer followed by quiescent and finally necrotic cells.

**Figure 5:**
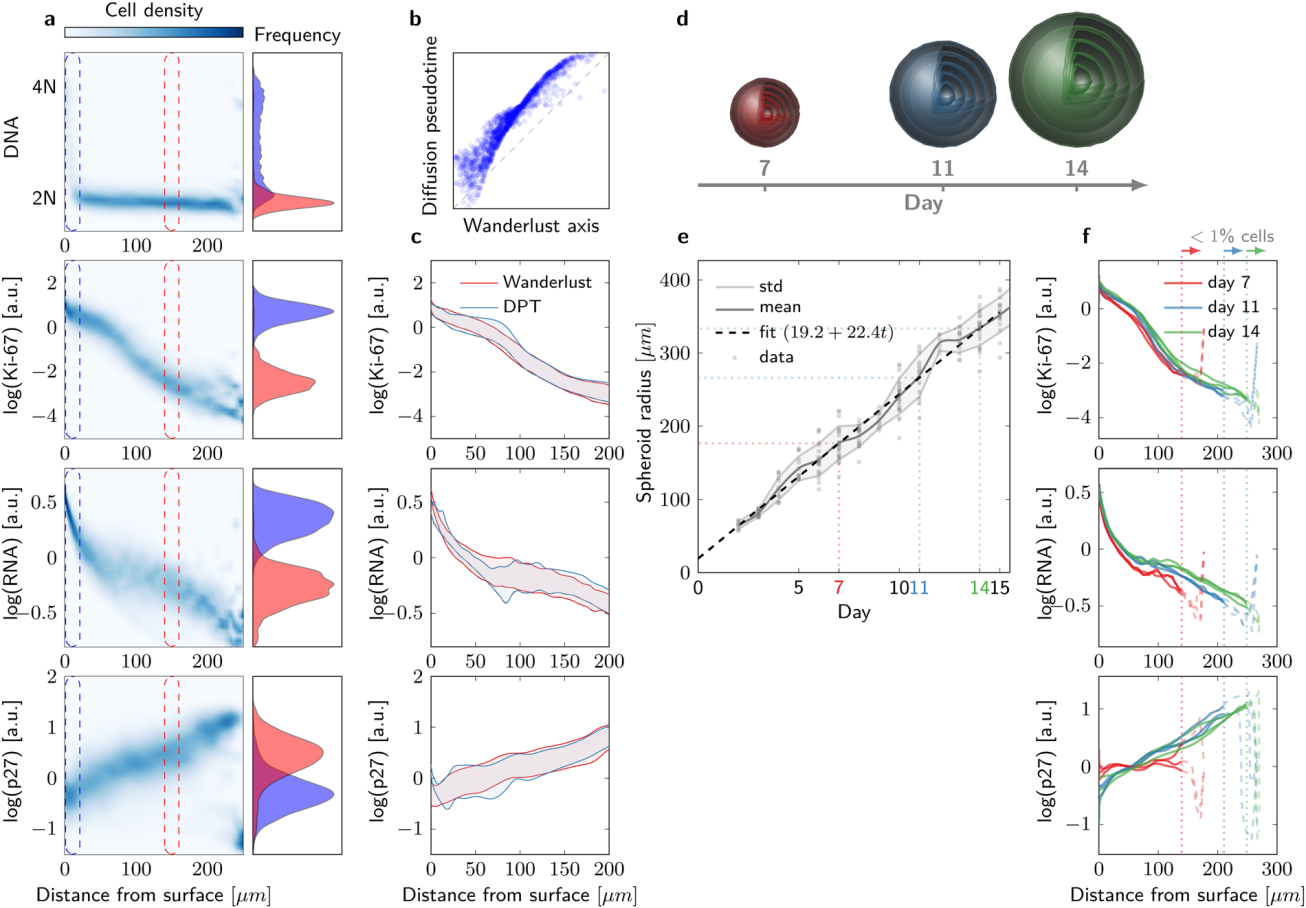
Studying cellular composition in spheroids of HCT116 cells with MAPiT. (**a**) Marginal signal densities $p(y| x)$ of indicated markers related to the distance from the surface, as obtained by MAPiT. Signal frequencies at the outermost layer and at 150 $\mu m$ distance from the spheroid surface, as indicated by the dashed rectangles, are shown for an exemplary 11-day-old spheroid. (**b**) Comparison of single-cell positions in pseudotime, as obtained by Wanderlust or DPT algorithms. (**c**) MAPiT robustly reconstructs cell positions, irrespective of the pseudotime algorithm used. Ranges show $50 \%$ confidence intervals of the signal intensities obtained from transforming Wanderlust and DPT pseudotime data. (**d**) Schematic of spheroid growth. (**e**) Spheroid growth follows a linear growth model. Microscopically obtained data shows spheroid radius over 15 days after seeding from $n=23$ spheroids in three independent replicates. (**f**) Median profiles for Ki-67, RNA and p27, as obtained by MAPiT, were conserved throughout spheroid sizes (median of $n=3$ spheroids, in three independent replicates). Notable deviations towards the ends of the profiles (dashed) and thus within the centre of the spheroids arise from inaccuracies due to the low number of cells available for analysis at these locations.

The DNA content of cells in the outermost spheroid layers exhibited a bimodality typically for proliferating cells with subpopulations in G1 (2N), S and G2/M (4N) phases (Fig. 5a). In contrast, the majority of cells in the inner layers remain in a quiescent G1/G0 (2N) state. The distinct distributions of additional markers, Ki-67, p27 and RNA content corresponded to this pattern (Fig. 5a).

We then studied the robustness of MAPiT, by comparing its performance to recover the spatial position using different pseudotime algorithms, markers and spheroid sizes. We first used the Wanderlust and Diffusion Maps algorithms^4,5^ to obtain a pseudotemporal ordering. The order of cells was largely conserved in both algorithms (Fig. 5b, monotonously increasing data points), however the pseudotime values were clearly different between both algorithms (Fig. 5b, deviation from the diagonal). Transforming the DPT and Wanderlust axis to a distance scale with MAPiT resulted in almost identical spatial profiles of Ki-67, RNA and p27 (Fig. 5c), thereby proving that MAPiT results are not affected by the pseudotime algorithm. MAPiT should likewise provide identical marker trajectories unaffected by the choice of markers used to generate the pseudotime order. To validate this, we conducted "leave one out cross validation", where we took subsets of the markers as input to the Wanderlust and DPT algorithms, and compared the results obtained by MAPiT. MAPiT robustly provided correct distance profiles of all markers in all combinations, demonstrating that MAPiT is not influenced by the choice of inputs to the pseudotime algorithms (Fig. S1). Thus, MAPiT is not affected by the choice of markers or pseudotime algorithms, provided that the order of cells on the pseudotime scale reflects the sequence or directionality of the biological processes.

Spatial reconstruction by MAPiT also provides scope to significantly accelerate high throughput studies that make use of advanced 3D culture-based cell screens, such as spheroid based viability assays and drug effect screens. Indeed, spheroids are considered superior models in comparison to conventional cell cultures^24^, but spatiotemporal analyses still require cumbersome and manual labour-intensive work for the analysis of cross-sectional slices. MAPiT performed reliably in 3D reconstruction when processing flow cytometry data from dissociated spheroids of different sizes and ages, based on Ki-67, RNA and p27 amounts (Fig. 5d–f). The profiles for Ki-67, RNA and p27 were conserved throughout spheroid sizes, indicating a dependence of these markers solely on the distance from the surface (Fig. 5f) and therefore their suitability for spatial reconstruction. Overall, robust spatial markers together with MAPiT therefore allow the rapid and versatile reconstruction of spheroids, providing a basis for studying spatiotemporal changes in other measurable variables.

## Discussion

In summary, MAPiT provides a solution to a fundamental problem, namely the transformation of pseudotime to real-time or the true scale of a biological process. For example, snapshot single-cell data of cell populations can be converted to extract real-time kinetics of cellular processes and responses, which otherwise could only be obtained by live-cell microscopy, which is more complex, time consuming and limited by the availability of suitable live-cell reporters. By reconstructing spatial and temporal spheroid compositions from single-cell data, MAPiT provides insights to the evolution of cellular heterogeneity within tumour-like microenvironments and allows to understand how responsiveness to therapeutics manifests within spheroidal environments. Since MAPiT provides the means to not only employ flow cytometric data but data from any other single-cell based high-throughput multiplex measurement, such as CyToF or single-cell RNA-seq, it provides a foundation for high-throughput and high-content studies of 3D-spheroid models by recovering the spatial information lost during spheroid dissociation. By extension, applying MAPiT to other single-cell snapshot data, such as single-cell transcriptomics and proteomics data, might significantly improve the inference of complex regulatory processes and networks by recovering real temporal and spatial dynamics.

In the present paper we apply MAPiT to two examples where we obtained the distribution on the desired scale by theoretical view on the source of heterogeneity. Constructing a target distribution for other processes i.e. the most common application of pseudotime single-cell analysis on stem cell differentiation, might be more challenging.

In the case of differentiation process, it might be necessary to not only measure cellular components for single cell analysis, but also cell death and cell division rates at every stage of the process (further discussed in the Supplementary Information). A time scale separation argument can be used to regard cell death and cell division as processes perpendicular to the cell differentiation. A suitable choice of markers may then allow inference of cell death and division rates from single cell data. We envision that combination of ergodic theory and mathematical models (as discussed in the Supplementary Information) to map single-cell differentiation data with MAPiT on a real time scale. Label data (e.g. sampling timepoints) may likewise be used to support or validate MAPiT results. However, knowing sources and sinks along the process like cell death or cell division is critical for obtaining the right real-time distribution and subsequent correct transformation with MAPiT.

Recently, pseudotime algorithms were further developed to robustly recognise also branching processes in differentiation pathways^8,25,26^, providing scope to apply MAPiT to study differentiation dynamics in individual branches. MAPiT could be applied to all paths from the root to the end points on the respective branches treating other the flow of cells into other branches as sinks.

Overall, MAPiT is a robust and universal tool to recover temporal or spatial cellular trajectories from high-throughput, high-dimensional single-cell experiments. MAPiT can be combined with pseudotime algorithms, and a MATLAB implementation is available through GitHub (https://github.com/karstenkuritz/MAPiT)^16^.

## Materials and Methods

### Calculation of pseudotime

Cells were ordered in pseudotime in MATLAB R2017b (MathWorks, Natick, MA, USA) using two different algorithms: Wanderlust^5^ and DPT^4^. The algorithms were run with 10000 randomly chosen cells. Prior to performing pseudotime analysis, doublets, dead cells and outliers were gated out. Both algorithms require a user defined set of root cells for constructing the pseudotime trajectories. For the cell cycle analysis a set of cells in the centre of the G1 population, with low DAPI and geminin signal, was chosen. For the spheroid analysis cells with high Ki-67 and high RNA signal which are known to be located at the surface of the spheroids were chosen.

### MAPiT theory

MAPiT is based on the measure-preserving transformation which states that one must conserve the area under the curve when transforming a probability distribution to another scale.

Consider a measure space $(X,{\mathscr{L}},\lambda )$, where $x$ is a set, ${\mathscr{L}}$ is a $\sigma -\,{\rm{ring}}\,$ of measurable subsets of $X$, and $\lambda$ is the measure. Given a map $\tau$ from a measure space $(X,{\mathscr{L}},\lambda )$ to a measure space $(Y,{\mathscr{S}},\mu )$, $\tau$ is called measurable if $A\in S$ implies ${\tau }^{-1}(A)\in L$. Given that $\tau$ is measurable, $\tau$ is called measure-preserving if $A\in S$ implies $\lambda ({\tau }^{-1}(A))=\mu (A)$. We denote the pseudotime values with $s\in [0,1]$, real-time scale with $x\in [0,T]$ and measured signals with $y\in {\mathbb{R}}$. Based on the general definition of a measure-preserving map $\tau$, the transformation $\tau :s\to x$ of a distribution of cells in pseudotime ${p}_{s}(s)$ to the distribution of cells on the real-time scale ${p}_{x}(x)$ reads

1 $${p}_{x}(x)=\left|\frac{{\rm{d}}{\tau }^{-1}(x)}{{\rm{d}}x}\right|\ {p}_{s}({\tau }^{-1}(x)),$$

2 $${P}_{x}(x)={P}_{s}({\tau }^{-1}(x)).$$

The mapping $\tau :s\to x$ from pseudotime to real-time was obtained by solving equation (2) for $\tau$, which then depends on the cumulative distributions of cells on both scales

3 $${\tau }^{-1}(x)={P}_{s}^{-1}\left({P}_{x}(x)\right),\,{\rm{or}}\,$$

4 $$\tau (s)={P}_{x}^{-1}\left({P}_{s}(s)\right).$$

Thus, by definition, the transformation $\tau$ requires knowledge of the distribution (or cumulative distribution) of cells on the pseudotime scale and the desired scale. If these distributions are positive (larger zero) over their support, then the cumulative distributions are monotonically increasing and the inverse exists. Once the mapping $\tau$ is known, one can apply the transformation to the joint densities of pseudotime and the observed quantities ${p}_{s}(s,y)$ to obtain the desired joint distribution of the true scale $x$ and measured markers $y$

5 $${p}_{x}(x,y)=\left|\frac{{\rm{d}}{\tau }^{-1}(x)}{{\rm{d}}x}\right|\ {p}_{s}({\tau }^{-1}(x),y).$$

Furthermore, we calculate the conditional or marginal density ${p}_{x}(y| x)$, which might be more informative, by normalizing the joint distribution with the real-time distribution

6 $${p}_{x}(y| x)={p}_{x}(x,y)/{\int }_{{\mathbb{R}}}{p}_{x}(x,y)\ {\rm{d}}y.$$

Distributions in pseudotime for spheroid data were obtained by kernel density estimation on pseudotime values ${s}_{i}$, using a Gaussian kernel with reflecting boundary at $s=\{0,1\}$,

7 $$\begin{array}{cc}{p}_{s}(s) & =\frac{1}{N}{\sum }_{i=1}^{N}({\mathscr{N}}(s|{s}_{i},{h}_{s})+{\mathscr{N}}(-s|{s}_{i},{h}_{s})+{\mathscr{N}}(2-s|{s}_{i},{h}_{s}))\\  & =\frac{1}{N}{\sum }_{i=1}^{N}{{\mathscr{N}}}_{r}(s|{s}_{i},{h}_{s}).\end{array}$$

Density in pseudotime for cell cycle data was estimated by kernel density estimation with linked boundary conditions to account for doubling of cell density during cell division^27^. Joint densities were calculated as sum of the product of the individual kernels

8 $${p}_{s}(s,y)=\frac{1}{N}{\sum }_{i=1}^{N}\left[{{\mathscr{N}}}_{r}(s| {s}_{i},{h}_{s})\ {\mathscr{N}}(y| {y}_{i},{h}_{y})\right].$$

Bandwidths ${h}_{s}$ and ${h}_{y}$ were derived from Silverman’s rule^28^.

### Distributions on the real scales

The distributions on the real scales can either be derived from theoretical considerations or from empirical measurements (discussed in Supplementary Information).

#### Cell density with respect to cell age in proliferating populations

For analysis of cell cycle-dependent processes with single-cell measurements, MAPiT requires the distribution of cells related to their cell cycle stage or equivalently their age $a$. Cell age, refers to the time since cell birth via cytokinesis. Our analysis is based on the following assumptions: (1) population is unperturbed and in its exponential growth phase, (2) no cell death in the population, (3) cell cycle progression is homogeneous. We thus restricted our analysis to unperturbed cell populations in their exponential growth phase with growth rate $\gamma$ and cell cycle length $T$ related by $\gamma =\frac{{\rm{ln}}\,2}{T}$. In such a case, the theoretical steady state age distribution of a cell population is given by^19^:

9 $${p}_{a}(a)=2\gamma {{\rm{e}}}^{-\gamma a}$$

The cumulative distribution and its inverse can be obtained in closed form:

10 $${P}_{a}(a)=2\left(1-{{\rm{e}}}^{-\gamma a}\right),$$

11 $${P}_{a}^{-1}(y)=-\frac{1}{\gamma }{\rm{ln}}\,\left(1-\frac{y}{2}\right).$$

Thus, in case of an unperturbed cell population it is sufficient to know the growth rate of the population to recover cellular age with MAPiT and thus obtain the temporal changes related to cell cycle progression of measured markers from one single-cell experiment. We verified all assumptions by live cell imaging. In addition, light scattering characteristics in the flow cytometric datasets were used to probe the population for cell death.

#### Cell density in tumour cell spheroids with respect to distance from surface

Cell density depending on the distance from the surface was obtained from sphere geometry (Fig. 2b, Supplementary Information Fig. S2). Our analysis is based on the following assumptions: (1) Spheroids are radial symmetric, (2) all cells in the spheroid have the same size. We verified both assumptions by visual inspection of whole spheroids and spheroid slices. The volume of a sphere with radius $r$ is given by

12 $${V}_{S}(r)=\frac{4}{3}\pi {r}^{3}.$$

The volume of a spheroid with necrotic core with radius ${r}_{N}=\max (r-{d}_{N},\ 0)$ equals

13 $${V}_{M}(r,{r}_{N})={V}_{S}(r)-{V}_{S}({r}_{N}),$$

with ${d}_{N}$ being the distance from the surface where the necrotic core begins. The volume of a spherical shell at distance $x$ from the surface of the spheroid with necrotic core is then given by

14 $$V(x)={V}_{M}(r,{r}_{N})-{V}_{M}(r-x,{r}_{N}).$$

Normalising (14) with the total spheroid volume ${V}_{M}$ results in the normalised volume with respect to the distance to the surface of the spheroid which represents the cumulative distribution of cells related to the distance from the surface

15 $${P}_{X}(x)=\frac{V(x)}{{V}_{M}(r,{r}_{N})}=\frac{{r}^{3}-{(r-x)}^{3}}{{r}^{3}-{r}_{N}^{3}}.$$

MAPiT is furthermore based on the probability density function and the inverse of the cumulative distribution which can be calculated analytically

16 $${p}_{X}(x)=3\frac{{(r-x)}^{2}}{{r}^{3}-{r}_{N}^{3}},$$

17 $${P}_{X}^{-1}(y)=r-\sqrt[3]{y({r}_{N}^{3}-{r}^{3})+{r}^{3}}.$$

Spheroid radius $r$ and the radius of first appearance of a necrotic core ${r}_{N}$ were inferred from experiments, described in more detail in the Supplementary Information. For the present study we used ${r}_{N}=270\ \mu m$ and a spheroid radius based on the linear regression

18 $$r(t)=19.2+22.4t$$

for spheroid growth as shown in Fig. 5e.

### Cell culture

The human colon carcinoma cell line HCT116 was obtained from the Banca Biologica e Cell Factory of the IRCCS Azienda Ospedaliera Universitaria San Martino in Genoa (ICLC HTL95025). The geminin expressing non-small-cell lung cancer NCI-H460 cells have been described previously^13^. NCI-H460/geminin cells were maintained in RPMI 1640 medium (Gibco, 21875034) supplemented with 5% heat-inactivated fetal calf serum (FCS, Pan - Biotech GmbH; P303309) and HCT116 cells were cultured in RPMI 1640 medium with 10% heat-inactivated FCS at 37 ${}^{\circ }$C in a humidified incubator with 5% CO${}_{2}$. For generation of tumour cell spheroids, cells were transferred into Terasaki multiwell plates (Greiner bio-one; 653180) in a volume of 25 $\mu l$ RPMI 1640 medium with a concentration of 4000 cells/ml. Thereafter, the plates were inverted to allow spheroid formation at the bottom of the emerging hanging drops and placed in humid chambers located in the incubator. Two to three days after seeding, formed spheroids were transferred to agarose-coated 96-well cell culture plates (Greiner bio-one; 655180 coated with 1.5% agarose (Carl Roth GmbH & Co. KG; 3810.3) in RPMI 1640 medium). Spheroid growth was monitored with an EVOS FL Cell Imaging System (Thermo Fisher Scientific Inc.) and spheroid diameters were determined from generated pictures using the Fiji software (distribution of ImageJ^29^).

### Time-lapse imaging

NCI-H460/geminin cells were imaged for their total cell cycle length and length of G1 or S/G2/M phases by time-lapse fluorescence microscopy using the Cell Observer system (Carl Zeiss, Oberkochen, Germany) equipped with a humidified imaging chamber at 37 ${}^{\circ }$C and 5% CO${}_{2}$. Randomly chosen cells were manually tracked for at least one full cell cycle and geminin signal intensity was recorded. Cell trajectories were obtained using the Tracking Tool (tTt) and qTfy for single-cell tracking and quantification of cellular and molecular properties in time-lapse imaging data^30^. For comparison to MAPiT derived results, background signal ${\theta }_{1}$ of cell trajectories and scaling factor ${\theta }_{2}$ for single-cell trajectories were chosen to maximise the log-likelihood between MAPiT density and individual traces,

19 $$\mathop{max}\limits_{\theta }\mathop{\sum }\limits_{i=1}^{N}\mathop{\sum }\limits_{k=1}^{{K}_{i}}{\rm{\log }}\,\left[p({t}_{k},({y}_{i}-{\theta }_{1}){\theta }_{2})\right],$$

where $p$ is the density obtained from MAPiT and ${y}_{i}$ are live cell imaging values for geminin of cell $i$ at time-points ${t}_{k}$ after cell division.

### Generation of spheroid sections and immunofluorescence staining for Ki-67

To generate spheroid sections, spheroids were fixed with 4% paraformaldehyd for 10 min at room temperature. Thereafter, spheroids were washed three times with PBS and finally kept in a sucrose solution (30% sucrose (Carl Roth GmbH & Co. KG; 4661) in PBS) at 4 $${}^{\circ }$$C. After 48 h, the sucrose solution was removed and replaced with Tissue Freezing Medium (A. Hartenstein GmbH; TTEK). Such embedded spheroids were stored at $$-20$$$${}^{\circ }$$C and finally cut into 10 $$\mu m$$ slices with a CM30505 cryostat. Generated sections were mounted on Polysine Microscope Adhesion Slides (Thermo Fisher Scientific Inc.; 10219280), fixed with 4% PFA for 10 min at room temperature (RT) and washed twice with PBS for 5 min. Thereafter, permeabilization was carried out with 0.1% Triton X-100 in PBS for 10 min before the slices were incubated with blocking solution (5% FCS and 0.1% Triton X-100 in PBS) for 30 min at RT. Incubation with the primary antibody against Ki-67 (1:400, Cell Signalling Technology; #9449) was carried out in blocking solution for 1 h at RT. Thereafter, sections were washed two times with blocking solution before they were incubated with the secondary antibody Alexa Fluor 647-conjugated goat anti-mouse IgG (1:500, Thermo Fisher Scientific Inc.; A-21236) solved in blocking solution for 1 h at RT in the dark. Next, sections were washed with blocking solution and incubated with DAPI (1 $$\mu $$g/ml in PBS, Thermo Fisher Scientific Inc.; D1306) to stain DNA for 10 min at RT before they were covered with coverslips using Fluoromount-G^®^ (SouthernBiotech; 0100-01). Samples were dried and fluorescence was analysed with a LSM 710 laser scanning microscope (Carl Zeiss, Oberkochen, Germany) and the blue edition of the ZEN software.

### Flow cytometric analysis

Tumour cell spheroids were dissociated with trypsin/EDTA (Gibco; 59418C) and single cells were fixed with 4% PFA in PBS for 10 min. Thereafter, permeabilization was carried out with 90% ice-cold methanol in PBS for 30 min on ice. Subsequently, cells were washed two times with a washing solution (5% FCS, 0.05% BSA (Sigma-Aldrich; A2058), 0.02% NaN${}_{3}$ (Carl Roth GmbH & Co. KG; Ca4221) in PBS) before they were incubated with the primary antibodies diluted in washing solution for 1 h at RT (anti-Ki-67, Cell Signalling Technology; #9449 and anti-p27, Cell Signalling Technology; #3686). Next, cells were washed and incubated with the respective secondary antibodies, Alexa Fluor 488-conjugated goat anti-rabbit IgG and Alexa Fluor 647-conjugated goat anti-mouse IgG diluted in washing solution for 1 h (1:100, Thermo Fisher Scientific Inc.; A11008 and 1:500, Thermo Fisher Scientific Inc.; A21236). After washing, cells were incubated with 100 $\mu l$ Hoechst 33342 solution (10 $\mu g/ml$ Hoechst 33342 (Thermo Fisher Scientific Inc.; H3570) in PBS with 0.05% BSA and 0.02% NaN${}_{3}$) for 1 h at 37 ${}^{\circ }$C, followed by the addition of 5 $\mu l$ Pyronin Y (stock solution: 100 $\mu g/ml$ solved in ddH${}_{2}$O, Sigma-Aldrich; 83200) and incubation for 15 min at 37 ${}^{\circ }$C. Finally, cells were pelleted, dissolved in PBS and fluorescence was measured by flow cytometry with the MACSQuant analyser 10. NCI-H460/geminin cells were analysed as described in^13^.

### Data size and statistical reporting

This section reports statistics of the described experiments. We abbreviate the number of biological replicates, representing replicates under the same protocol but in different experiments, with ${n}_{b}$. Technical replicates abbreviated by ${n}_{t}$ are obtained in identical experimental conditions, e.g. flow cytometric measurements of ${n}_{t}=3$ spheroids from the same culture. Furthermore, we denote the samples size, e.g. number of single cells in a single cell experiment, by ${n}_{s}$.

Flow cytometry measurements of HCT116 spheroids were performed in three independent experiments (${n}_{b}=3$). Each experiment was carried out with three spheroids (${n}_{t}=3$). A samples size of ${n}_{s}=$10,000 cells was subsequently used for analysis with MAPiT. Ki-67 profiles in sliced spheroids are reported for one representative spheroid, with few defects caused by the slicing procedure (${n}_{b}={n}_{t}=1$). Nine profiles throughout the spheroid resulted in ${n}_{s}=18$ rim-to-core profiles. Cell cycle analysis of flow cytometric measurements was done with ${n}_{s}=$10,000 cells in one representative experiment (${n}_{b}={n}_{t}=1$). Live-cell imaging was done in one experiment (${n}_{b}={n}_{t}=1$) wherein we recorded the geminin intensity of ${n}_{s}=12$ randomly chosen cells over one full cell cycle.

Signal intensities in the spheroid experiments were normalised to the median intensity of all cells up to a distance of $150\ \mu m$ from the surface. MATLAB code for comparison of MAPiT analysis with imaging data is available in the MAPiT repository (https://github.com/karstenkuritz/MAPiT)^16^.

## Supplementary information

Supplementary Information.
